# *Colletotrichum orbiculare* strains distributed in Japan: race identification and evaluation of virulence to cucurbits

**DOI:** 10.1270/jsbbs.22011

**Published:** 2022-08-03

**Authors:** Hiroki Matsuo, Yasuhiro Ishiga, Yasuyuki Kubo, Yosuke Yoshioka

**Affiliations:** 1 Graduate School of Life and Environmental Sciences, University of Tsukuba, Tsukuba, Ibaraki 305-8572, Japan; 2 Faculty of Life and Environmental Sciences, University of Tsukuba, Tsukuba, Ibaraki 305-8572, Japan; 3 Faculty of Agriculture, Setsunan University, Hirakata, Osaka 573-0101, Japan

**Keywords:** anthracnose, *CsSGR*, Cucurbitaceae, nucleotide-binding leucine-rich-repeat receptors, pathogenicity

## Abstract

The pathogen *Colletotrichum orbiculare* is causal fungus of cucurbit anthracnose. Multiple races have been identified in the United States, suggesting that it is necessary to cultivate suitable resistant cultivars and breed new cultivars with the most suitable resistance gene. This study examined the pathogenicity and virulence of 20 strains in Japan to clarify the existence of races and virulence differences. Based on the symptoms on inoculated cotyledons and true leaves of watermelon, we could evaluate the compatibility of each strain to each host cultivar. Our analysis based on the reaction to the host cultivar harboring the resistance gene *Ar-1* (*Cla001017*) revealed the existence of three races in Japan. An alarming result was that a race that overcame *Ar-1*, which is a target gene in current watermelon breeding in Japan, is present in Japan. The cucumber and melon host cultivars showed diverse symptoms, whereas a squash cultivar was resistant to all strains. Three strains caused severe damage even to the most resistant cucumber cultivar ‘Ban Kyuri’ and resistant cultivars harboring *Cssgr*, a well-known gene conferring loss-of-susceptibility resistance. Screening genetic resources for novel resistance genes using strains with high virulence is of vital importance for watermelon, cucumber, and melon production.

## Introduction

Cucurbit anthracnose, caused by a fungal pathogen *Colletotrichum orbiculare* (Berk. et Mont.) Arx (syn. *C. lagenarium* [Pass.] Ellis et Halst.), reduces the yield and quality of agricultural production in cucurbits, such as watermelon (*Citrullus lanatus* [Thunb.] Matsum. & Nakai), cucumber (*Cucumis sativus* L.), and melon (*Cucumis melo* L.), especially in warm and humid regions of the world. *Colletotrichum orbiculare* is a hemi-biotrophic pathogen that infects hosts via biotrophic and necrotrophic stages (O’Connell *et al.* 2012). In the biotrophic stage, *C. orbiculare* penetrates and invades the cytoplasm of host cells, and in the subsequent necrotrophic stage it degrades host tissues and subsequently causes host plants to wilt. During this infection process, the fungus causes symptoms on the host organs and spreads throughout the plant aboveground organs, including stems, leaves, and fruits (Cannon *et al.* 2012).

In general, genetically conferred host resistance based on innate immunity is the best for controlling fungal disease. The pattern recognition receptors (PRRs) control innate immunity by recognizing effectors and general patterns of microbes, then activating a defense response (e.g., hypersensitive reaction). Genes encoding PRRs are often identified as causal genes of cultivar-specific resistance (*R* genes). The representative PRRs are proteins containing the leucine-rich repeat domain and include receptor-like proteins/kinases and nucleotide-binding leucine-rich-receptors (NLRs). The relationship between *R* genes and effectors has been defined as race-specificity based on the gene-for-gene theory (Flor 1971). That is, the compatibility between pathogen and host plant depends on the genotype of the avirulence (*Avr*) gene in the pathogen and *R* gene in the host. The gene-for-gene theory generally explains the interaction between a plant species and its pathogen that infects via biotrophic or hemi-biotrophic stages. Race specificity is considered to arise through the arms-race evolution between effectors and *R* genes, and *Avr* genes are well known as a subset of effectors (Jones and Dangl 2006).

The multiple races (1–7) of *C. orbiculare* have been identified on the basis of their pathogenicity or virulence on cucurbits including watermelon, cucumber, melon, and squash in the United States (Dutta *et al.* 1959, Goode 1956, 1958, Jenkins *et al.* 1964). Wasilwa *et al.* (1993) re-evaluated the pathogenicity of *C. orbiculare* strains and concluded that there were only two races in the United States; race 1 is highly virulent on cucumber, melon, and some watermelon cultivars, whereas race 2 is moderately virulent on cucumber but highly virulent on most watermelon cultivars. In the study by Wasilwa *et al.* (1993), the watermelon cultivars ‘Charleston Gray’ and ‘Crimson Sweet’ were used as differential cultivars because their responses were the most significant between races: they were resistant to race 1 and susceptible to race 2. Jang *et al.* (2019) reported that a non-synonymous mutation in *Cla001017*, which encodes a NLR, causes resistance to race 1. This mutation is conserved among various watermelon cultivars resistant to race 1, including ‘Charleston Gray’ and ‘Crimson Sweet’ (Jang *et al.* 2019). Thus, the gene with this mutation is considered to be the causal gene of *Ar-1* in the watermelon gene list reported by Guner and Wehner (2003). The resistant genetic resources and cultivars harboring a resistance allele (*Ar-2*) to race 2 were also reported in watermelon (Boyhan *et al.* 1994, Guner and Wehner 2003). In cucumber, PI 197087 is resistant to anthracnose (Barnes and Epps 1952, 1954), and its resistance locus (*cla*) has been used widely in breeding for nearly 60 years in the United States. *STAYGREEN* (*CsSGR*) was identified as a causal gene for *cla* to race 1 in an inbred line ‘Gy14’ derived from PI 197087 (Pan *et al.* 2018). This recessive allele (hereafter referred to as *Cssgr*) carries a loss-of-susceptibility mutation and suppresses chlorosis under *C. orbiculare* infection. In addition, it confers broad-spectrum resistance to downy mildew (*Pseudoperonospora cubensis*) and angular leaf spot (*Pseudomonas syringae* pv. *lachrymans*) (Wang *et al.* 2018). However, PI 197087 and its derivatives ‘Poinsett 76’, ‘Gy14’, and ‘H19’ were susceptible to race 1 but moderately resistant to race 2 (Wasilwa *et al.* 1993). There appears to be a discrepancy between the results of Wasilwa *et al.* (1993) and Pan *et al.* (2018) regarding the response of *Cssgr* derived from PI 197087 to race 1.

The race differentiation of *C. orbiculare* in the United States suggests that it is necessary to assess existence of races in other regions and to cultivate suitable resistant cultivars and breed new cultivars with the most suitable resistance gene. To date, races of *C. orbiculare* have not been examined in detail in Japan. However, our previous study indicated the possibility of diversity of pathogenicity and virulence to cucumber and watermelon in *C. orbiculare* strains collected in Japan (Matsuo *et al.* 2022a, 2022b). In this study, we examined the pathogenicity and virulence of 20 *C. orbiculare* strains distributed in Japan to clarify the existence of races and difference of virulence by conducting qualitative and quantitative assays on four cucurbit host species. In addition, we propose a new set of differential cultivars that are available in Japan for characterization of *C. orbiculare* races, because several cultivars and/or accessions used by Wasilwa *et al.* (1993) in the United States cannot be easily obtained in Japan.

## Materials and Methods

### Plant and fungal materials

We used 16 cultivars/lines of four cucurbit hosts preserved in the Genebank of the National Agriculture and Food Research Organization, Japan (NARO Genebank) and in the University of Tsukuba: PI 197087 (C01), ‘Poinsett’ (C02), ‘Ban Kyuri’ (C03), a Beit-Alpha type fixed line (C04), ‘Tokiwa’ (C05), ‘Marketer’ (C06), ‘Model’ (C07), ‘SMR 58’ (C08) of cucumber, ‘Earls Favorite Harukei 3’ (M01), ‘Edist 47’ (M02) of melon, ‘Sugar Baby (Cocomero)’ (W01), ‘Charleston Gray’ (W02), ‘Sugar Baby A’ (W03) and two commercial F1 cultivars (W04, W05) of watermelon, and one commercial F1 cultivar (S01) of squash.

We assayed 20 strains of *C. orbiculare* originated in Japan: 15 strains preserved in the NARO Genebank, one strain kindly provided by Nanto Seed Co., Ltd (Kashihara, Japan), and 4 strains isolated as single colonies derived from symptomatic watermelon and cucumber in 2021 (Table 1). Strains were cultured on potato dextrose agar (PDA; Nissui Pharmaceutical Co., Ltd., Tokyo, Japan) at 25°C under light. Strains that did not form conidia by this method were cultured on PDA under UV light at 25°C or on oatmeal agar (Becton, Dickinson and Company, Franklin Lakes, NJ, USA) at 25°C in the dark. Mycelial plugs with conidia were soaked overnight in 10% glycerol (Fujifilm Wako Pure Chemical Corporation, Osaka, Japan) and then stored at –80°C. For inoculum preparation, a mycelial plug was transferred onto PDA and incubated at 25°C under light for 10 days. The mycelium and spores were then suspended in deionized water and spread on other PDA plates, and the plates were incubated at 25°C under light for 5–7 days. Spores formed on the surface were suspended in deionized water and filtered through Kimwipe tissue (Nippon Paper Crecia Co., Ltd., Tokyo, Japan).

### Species identification of field isolates based on molecular phylogenetic analysis

DNA was extracted from mycelia of four field isolates (IT21-1, IT21-2, ST21-1, ST21-2) using the DNeasy Plant Mini Kit (Qiagen, Hilden, Germany) with minor modifications. The 5.8S nuclear ribosomal gene region with two internal transcribed spacers (ITS1-5.8S-ITS2) was amplified by PCR with ITS5 and ITS4 primers (White *et al.* 1990). The PCR cycling conditions described by Sato *et al.* (2012) were used with minor modifications. All amplification products were sequenced, and resulting sequences were then aligned and assembled by GeneStudio Inc. (Suwanee, GA, USA).

**Fig. 1.**
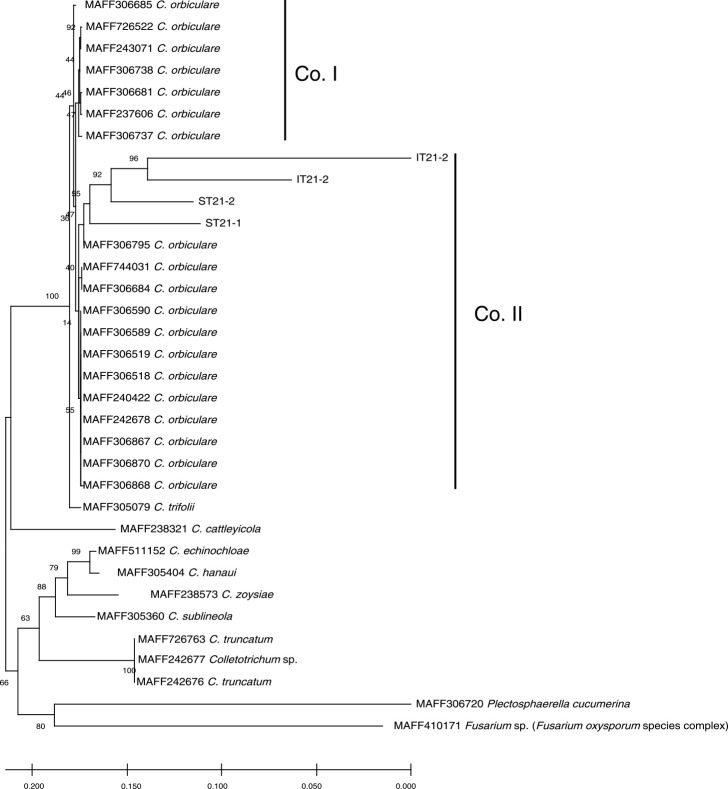
Phylogenetic tree illustrating relatedness of *Colletotrichum* species and field isolates, based on neighbor-joining analysis of the ITS1-5.8S-ITS2 rDNA region. Numbers at nodes are percentages of 1000 bootstrapped data sets that support specific branches. Bar = distance corresponding to two base changes per 100 nucleotide positions. Co. I and Co. II at the side of the tree indicate phylogenetic groups of *C. orbiculare*.

The sequences of the four field isolates were compared with those of 18 *C. orbiculare* strains and 9 *Colletotrichum* sp. strains (MAFF 305079, 238321, 511152, 305404, 238573, 305360, 726763, 242677, 242676) preserved at the NARO Genebank by phylogenetic analysis (Fig. 1). In addition, MAFF 306720, a causal fungus of cucumber anthracnose but not *C. orbiculare*, and MAFF 410171, a strain of *Fusarium* sp. (the *Fusarium oxyporum* species complex) from the NARO Genebank (Aoki *et al.* 2020) were used as an outgroup in this analysis; their sequences were obtained from the NARO Genebank website (https://www.gene.affrc.go.jp). Multiple sequence alignment of the data was initially carried out using MEGA X (Kumar *et al.* 2018). Phylogenetic trees, showing the relatedness between isolates, were constructed from a distance matrix by using the neighbor-joining method (Saitou and Nei 1987). The distance in the ITS1-5.8S-ITS2 region was determined by Kimura’s two-parameter model (Kimura 1980; Moriwaki *et al.* 2002). Confidence limits for the branches based on parsimony criteria were estimated by bootstrap analysis of 1000 replicates. In addition, online BLAST searches (https://blast.ncbi.nlm.nih.gov/Blast.cgi) were performed using the sequence of each isolate.

### Detection of polymorphism at *Cla001017* in watermelon and *CsSGR* in cucumber by using CAPS markers

Genomic DNA was extracted from cotyledons using the DNeasy Plant Mini Kit (Qiagen, Hilden, Germany) according to the manufacturer’s protocol with minor modifications. Genotyping of *CsSGR* was performed as described by Matsuo *et al.* (2022a). For *Cla001017* genotyping to determine whether each cultivar was wild type (susceptible) or mutant (resistant), we used a cleaved amplified polymorphic sequence (CAPS) marker designed with a 300-bp amplicon size by Primer3plus (https://www.bioinformatics.nl/cgi-bin/primer3plus/primer3plus.cgi) and Oligo calculator (http://www.ngrl.co.jp/tools/0217oligocalc.htm): forward primer 5ʹ-GCTTCCAAAATCTATTGTTTTGCT-3ʹ; reverse primer 5ʹ-TTTTTACTTCCAACACGCTCG-3ʹ. PCR mixtures (5 μL) contained template DNA of each sample, Blend Taq-Plus, 10× buffer, 2 mM dNTPs (Toyobo Co., Ltd., Osaka, Japan), autoclaved Milli-Q water, and primer mix. The PCR conditions were 94°C for 2 min; 35 cycles of 94°C for 30 s, 55°C for 30 s, 72°C for 30 s; and a final extension at 72°C for 3 min. Amplified DNA was digested with *Hind* III (Takara Bio Inc., Kusatsu, Japan) in 10× M buffer and autoclaved Milli-Q water at 37°C for 2 h. The digested products were electrophoresed in 8% polyacrylamide gel. The gel was stained with GelRed (0.03 μL/mL; Cosmo Bio Co., Ltd., Tokyo, Japan) and the bands were visualized under UV light. The *Cla001017* genotype of each plant was determined from the band size: the digested band (250 bp) is the mutant allele, and the undigested one (300 bp) is the wild-type allele.

### Detached cotyledon assay

Twenty strains of *C. orbiculare* were qualitatively evaluated for pathogenicity to cucumber and watermelon in the detached cotyledon assay. The cucumber cultivars assessed were C01 and C02, with homozygous *Cssgr*; C03, a resistant cultivar that is supposed to have different resistance genes (Matsuo *et al.* 2022a); and C04 and C05, two susceptible fixed lines. Two watermelon cultivars were also used: W02, which is homozygous for the resistant-type allele (*Ar-1*) of *Cla001017*, and W01, which is homozygous for the susceptible-type allele (hereafter referred to as *ar-1*). The cultivars resistant to race 2 identified by Boyhan *et al.* (1994) and Guner and Wehner (2003) were not included because these are not preserved in the NARO Genebank.

For each strain, four cotyledons of each cucumber and watermelon cultivar were tested. To make all watermelon cultivars germinate at the same time, the seeds were soaked overnight in 30°C water in a shaker. Then watermelon and cucumber seeds were placed in separate Petri dishes lined with wet filter paper (Toyo Roshi Kaisha, Ltd., Tokyo, Japan) and incubated at 28°C in the dark. Germinated seeds were transplanted into 38-mm-diameter plastic cell trays filled with moist culture soil (Nihon Hiryo Co., Ltd., Fujioka, Japan) and incubated at 28°C under a 12-h photoperiod in a climate chamber (Nippon Medical and Chemical Instruments Co., Ltd., Osaka, Japan). After cotyledon unfolding, the chamber temperature was set to 25°C for 8 days. Then, the cotyledon petioles were cut and planted in a Petri dish filled with 1/2 B5 medium (1.65 g/L Gamborg’s B5 medium salt mixture, 0.7 g/L agar powder for plant culture medium; all reagents were from Fujifilm Wako Pure Chemical Co., Ltd). The Petri dish lid was closed to maintain high humidity and the material was incubated overnight in the dark at 25°C. Next, 10 μL of inoculum was dropped onto each cotyledon surface six times at different points for cucumber and four or six times on watermelon. The concentration of each inoculum was adjusted so that lesions were formed on the cotyledon of susceptible cultivar C04 (the class of S described below). After inoculation, the cotyledons were kept in the dark at 25°C for 24 h. The lids were then opened, and the cotyledons were grown at 25°C in a 12-h photoperiod in the climate chamber.

Ten days after inoculation, the severest lesion on each cotyledon was evaluated and scored as 1, no lesion; 2, lesion ≤3 mm in diameter; or 3, lesion >3 mm in diameter. These scores were averaged for each cultivar, and the cultivar was classified as resistant (R; <1.5), moderately resistant (MR; 1.5–2.5), and susceptible (S; >2.5). Strains were then categorized into several groups based on the resistance levels of host cultivars of watermelon and cucumber separately.

### Seedling assay

Pathogenicity and virulence of *C. orbiculare* strains were also tested by conducting a seedling assay. Ten individuals per cucumber, melon, watermelon, and squash cultivar were tested. To make all watermelon accessions germinate at the same time, the seeds were soaked overnight in 30°C water in a shaker. The seed coat of watermelon accessions with a thick seed coat was scratched with a nail clipper before soaking. Then seeds of cucurbit materials were placed in Petri dishes lined with wet filter paper and incubated at 28°C in the dark for a day. Germinated seeds were transplanted into 38-mm-diameter plastic cell trays filled with moist culture soil and incubated at 28°C under a 12-h photoperiod in a climate chamber. After cotyledon unfolding, the chamber temperature was set to 25°C during the day (12 h) and 18°C at night for 18 days. Plants were placed inside of clear plastic boxes (60 per box; Akasaka Co., Ltd., Atsugi, Japan), and inoculum was sprayed on the upper surface of each true leaf (ca. 0.4 mL) from a spray bottle. The inoculum concentration was adjusted to 1.0 × 10^6^ spores/mL, and 0.5 μL/mL Tween-20 (Kanto Chemical Co., Inc., Tokyo, Japan) was added. The boxes were closed to maintain 100% relative humidity and kept in the dark for 24 h. The lids were then opened, and the plants were grown at 25°C under a 12-h photoperiod in the climate chamber.

The resistance of each inoculated plant was scored 1 week after inoculation. Scoring was based on infection severity (IS) levels: 0, no lesion; 1, a few small lesions (<3 mm in diameter); 2, several small lesions or a few medium lesions (3–5 mm); 3, many small lesions and a few to several medium lesions, or a few large lesions (>5 mm), development of chlorotic boundaries; 4, several medium lesions and a few large lesions, development of chlorotic boundaries; 5, many large lesions cover half of the true leaf or more than half of the true leaf is withered; 6, the true leaf is completely withered; 7, the whole seedling is wilting and dead.

## Results

The ITS1-5.8S-ITS2 sequences of the four strains, IT21-1, IT21-2, ST21-1 and ST21-2 isolated from watermelon fruit and cucumber leaf (Table 1), matched the rDNA region of *C. orbiculare* with over 94% of query cover according to a BLASTN search (IT21-1: HQ839785.1, query cover 94%; IT21-2: KT454386.1, query cover 95%; ST21-1: AJ301965.1, query cover 98%; ST21-2: AJ301965.1, query cover 98%). All *C. orbiculare* strains were placed in the same clade based on phylogenetic analysis using the sequence of the ITS1-5.8S-ITS2 rDNA region (Fig. 1). The *C. orbiculare* clade was further divided into Co. I and Co. II subgroups, and the four strains belonged to the Co. II subgroup (Fig. 1). These results indicated that all four strains were *C. orbiculare*.

In the detached cotyledon assay, 9 strains formed no lesion, and 11 strains formed small to large lesions in watermelon cultivars (Table 1). In contrast, all 20 strains formed small to large lesions on cotyledons of any of cucumber cultivars (Table 2), in particular 5 strains formed large lesions on all host cultivars (Table 2). Based on the resistance levels of host cultivars, the 20 strains were categorized into three groups (AW0–2) in watermelon (AW0: W01 and W02 were not S; AW1: only W01 was S; AW2: W01 and W02 were S) and seven groups (AC1–7) in cucumber (AC1: only C04 was S; AC2: C01 and C04 were S; AC3: only C05 was S; AC4: C01 and C04, C05 were S; AC5: C01 and C02, C4 were S; AC6: cucumber hosts except C03 were S; AC7: all cucumber hosts were S) (Tables 1, 2). There did not seem to be a strong relationship between the AW and AC groups, because each AW group included several strains belonging to different AC groups. For example, AW0 included strains belonging to AC1, AC2, and AC5–AC7 (Table 1). In addition, there was no association among the AW and AC groups, the clusters based on the molecular phylogenetic tree (Co. I and Co. II), and geographic origin (Tables 1, 2). Almost all strains infected not only the original host species but also other species, indicating that they have adapted as common pathogens to melon, cucumber, and watermelon.

Based on the results of the detached cotyledon assay, 17 typical strains in the AW0–2 and AC2–7 groups were selected, and the pathogenicity and virulence of each strain was further tested by conducting a seedling assay on four cucurbit species. The lowest IS score among all hosts was found in the S01 cultivar of squash, with no or a few small lesions in response to all strains (Table 3). Two melon cultivars tended to have high IS scores (>4.0) and were susceptible to almost all strains except for MAFF 240422 (Table 3). In contrast, there was wide variation in the IS scores of watermelon and cucumber cultivars (Table 3, 4).

The IS scores of all watermelon cultivars tended to be lower than those of cucumber and melon. W02 and W05, homozygous and heterozygous for the *Cla001017* mutant allele (*Ar-1*), respectively, showed lower IS scores on average than the remaining cultivars. The seedling and cotyledon assays gave consistent results. Among AW groups, AW0 did not cause severe symptoms in any watermelon cultivar, although there were significant differences in the IS score of several strains among cultivars (Table 3). In contrast, W01, W03, and W04 were severely infected by most AW1 strains, whereas W02 and W05 survived and showed lower IS scores. There were differences in virulence among strains of AW1: the IS scores of W02 and W05 were ≥3.0 (moderate resistance) for MAFF 306870 and ST21-2, while they were <3.0 for MAFF 242678, 306590, 306519, 726522, and 306737 (Table 3). Among AW2 strains, IT21-1 and IT21-2 caused wilting (IS score = 7) of nearly all watermelon hosts (Table 3). ST21-1 also caused wilting in W01, W03, and W04, but W02 and W05 exhibited small lesions and survived. These results indicate that the virulence and pathogenicity of ST21-1 to W02 and W05 was different from that of IT21-1 and IT21-2. The results of both cotyledon and seedling assays allowed us to identify and distinguish three *C. orbiculare* races in watermelon (Table 3). Results of detached cotyledon and seedling assays didn’t match necessarily in watermelon. Detached cotyledon assay is a high efficiency method but requires cutting cotyledon and damages plants before inoculation. This damage may enhance the susceptibility to *C. orbiculare* and other disease in watermelon cotyledon. Similar difference between detached cotyledon and true leaf assays was also observed in our previous work related to cucumber anthracnose resistance (Matsuo *et al.* 2022a). Therefore, seedling assay using young plants with one or two expanded true leaves is considered to be more accurate at least for cucurbit anthracnose.

**Fig. 2.**
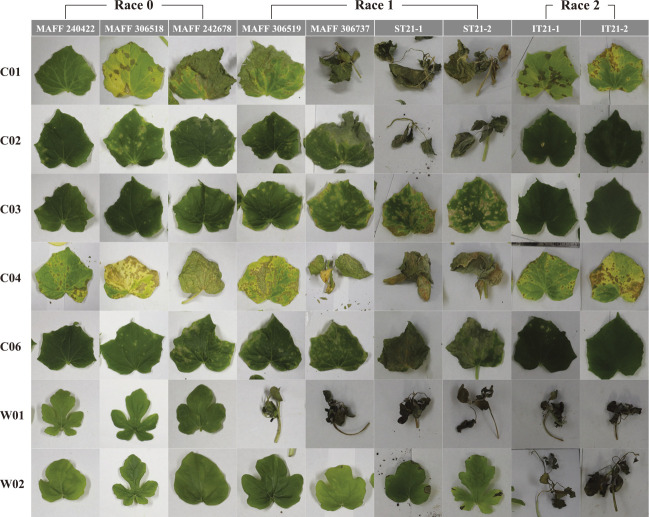
Variation of symptoms on true leaves of watermelon and cucumber in the seedling assay.

Among cucumber hosts, C03 showed the lowest IS score, on average, followed by C06, which is homozygous for *Cssgr*. Some results were inconsistent between the seedling and cotyledon assays. For example, MAFF 306681 and MAFF 243071 were categorized as AC7 and AC6, respectively, but their IS scores in the seedling assay were not high (Table 4). However, the remaining AC7 and AC6 strains tended to have higher IS scores. Among AC7 strains, MAFF 306737 and ST21-2 caused cucumber cultivars the most severe symptoms, with C03 and three cultivars homozygous for *Cssgr* showing IS scores >4.4 (Fig. 2, Table 4). A strain of AC2 had the lowest IS scores as expected, and caused medium lesions only for C04. Although the level of virulence was diverse in resistant cultivars (i.e., C03 and three cultivars homozygous for *Cssgr*), our data did not allow us to identify and distinguish different races in cucumber due to the continuous distribution of the values.

## Discussion

Race–cultivar specificity based on the gene-for-gene theory (Flor 1971) is generally explained as the compatibility between pathogen *Avr* or *avr* genes and the genotype of the host *R* gene (Collinge 2020). Previous studies attributed the resistance to *C. orbiculare* in cucurbits to *Ar-1* (*Cla001017*) and *Ar-2* of watermelon (Guner and Wehner 2003, Jang *et al.* 2019) and *cla* of cucumber (Pan *et al.* 2018, Wang *et al.* 2018). In the latest report of race classification in the United States (Wasilwa *et al.* 1993), watermelon cultivars ‘Charleston Gray’ and ‘Crimson Sweet’ and cucumber cultivars ‘H19’, ‘Gy14’, ‘Poinsett76’, and PI 197087 were used as differential cultivars. These watermelon cultivars harbor the *Ar-1* allele (Jang *et al.* 2019), and the cucumber cultivars harbor the *Cssgr* allele (Pan *et al.* 2018). The race can be identified by only the *Ar-1* genotype of watermelon because *Cssgr* is related to loss-of-susceptibility resistance and is not an *R* gene. In fact, the reaction of watermelon host to anthracnose strains can easily be evaluated via qualitative phenotypes: wilting or surviving (Fig. 2).

In watermelon, therefore, race 1 is incompatible with *Ar-1* and compatible with *ar-1*; race 2 can overcome *Ar-1* and thus is compatible with *Ar-1* and *ar-1*. Furthermore, we designated race 0, which is incompatible with both *Ar-1* and *ar-1* or an unknown resistance gene that all watermelon cultivars used in this study have. Otherwise, race 0 might be a group of *C. orbiculare* strains that does not adapt to watermelon. The groups AW0, AW1, and AW2 in this study basically correspond to watermelon anthracnose races 0, 1, and 2, respectively, with some exceptions. According to AW groups, we classified each strain into a race (Table 3). However, MAFF 242678 and 306590, which belong to AW1, were classified as race 0 because of lower IS scores (<5.0) of the cultivar homozygous for the susceptible-type allele (*ar-1*) in the seedling assay. Similarly, MAFF 306738, 306795, and 237606 of AW0 were classified as race 1 because of higher IS scores (>5.0) on the cultivar homozygous for *ar-1*. In addition, ST21-1 of AW2 was also classified as race 1 because of lower scores (<5.0) of the cultivar homozygous for the resistance-type allele (*Ar-1*).

By contrast, the cucumber host cultivars showed diverse IS scores regardless of the race defined by the watermelon resistance gene, especially in the cultivars homozygous for *Cssgr* (Fig. 2). All three cultivars homozygous for *Cssgr* were severely damaged (wilted) by ST21-1 and ST21-2 of the AC7 group, which had the highest virulence to cucumber (Table 4). Among cultivars homozygous for *Cssgr*, C06 (‘Marketer’) tended to show moderate resistance to the remaining strains of AC7, whereas C01 (PI 197087) was severely damaged (wilted) by three strains (MAFF 306870, 726522, 306737) (Table 4). Matsuo *et al.* (2022a) also reported that a cultivar homozygous for *Cssgr* was severely damaged by MAFF 306737.

We propose two hypotheses to explain the different reaction to Japanese anthracnose strains among cultivars homozygous for *Cssgr*. The *STAYGREEN* (*SGR*) gene is a vital regulator of the chlorophyll degradation pathway, an important event in leaf chlorosis (Kuai *et al.* 2018, Shimoda *et al.* 2016, Thomas and Ougham 2014). *SGR* expression is also crucial for pathogen infection and the development of chlorosis (Ishiga *et al.* 2015, Mecey *et al.* 2011). The *Cssgr* allele with a non-synonymous substitution in the *SGR* domain is responsible for the resistance phenotype, as its expression level does not increase during *C. orbiculare* infection, resulting in the inhibition of chlorosis (Pan *et al.* 2018, Wang *et al.* 2018). Mutations in the regulators of chlorosis, including genes related to the chlorophyll degradation pathway, may significantly influence disease resistance. Thus, as the first hypothesis, cultivars homozygous for *Cssgr* but susceptible to highly virulent strains of group AC7 may have other mutations in the coding region of *CsSGR* that promote chlorosis or have other independent allele(s) that inhibit *Cssgr* resistance and result in a susceptible phenotype with wilting.

Cucumber accession PI 197087 (C01) is a well-known donor of *Cssgr* in cucumber breeding (Pan *et al.* 2018, Wang *et al.* 2018). The accession WI 2757 has *Cssgr* derived from PI 197087 and minor QTL *cla3.1* contributing resistance to *C. orbiculare* race 1 in the United States (Pan *et al.* 2018). Like WI 2757, the derivative line of PI 197087 might have acquired other resistance factors in the breeding process. Thus, as a second hypothesis, strains that overcome the resistance of *Cssgr* evolved in the process of virulence differentiation, and genes other than *Cssgr* may enhance the resistance to such strains. In the case of downy mildew caused by *P. cubensis*, the recessive resistance gene *dm1* was identified (Barnes and Epps 1954), and numerous resistant cultivars carrying *dm1* were released and have been cultivated for decades. However, a new pathotype of *P. cubensis* emerged in 2004 and overcame the *dm1* resistance (Holmes *et al.* 2006, Thomas *et al.* 2017). It is now well known that several accessions harboring other resistance genes or *dm1* plus them exhibit strong resistance to the new pathotype of downy mildew (Wang *et al.* 2020). The resistance conferred by *dm1* is loss-of-susceptibility resistance, and *dm1* is identical to *Cssgr*. Thus, this situation with downy mildew suggests that our second hypothesis about variation of resistance among cultivars harboring *Cssgr* is quite conceivable.

The squash cultivar (S01) was resistant to all strains (Table 3). The causal agent of squash anthracnose was reported to be *Colletotrichum capsici* (Yaguchi *et al.* 1996). *Colletotrichum orbiculare* was also isolated from imported pumpkin fruits in Japan (Kobayashi and Kimishima 1997), but *C. orbiculare* has tended to be incompatible with the *Cucurbita* genus (Akai and Terasawa 1957, Kanno and Moriwaki 2000, Wasilwa *et al.* 1993). No strains used in this study infected S01, supporting the idea that *C. orbiculare* is not well adapted to *Cucurbita*.

In conclusion, we focused on the compatibility to *Ar-1* of watermelon and revealed the existence of anthracnose races 0, 1, and 2 in Japan. The diverse differentiation in the virulence to cucumber occurred independently of race in Japan. Therefore, it is important to select a *C. orbiculare* strain for use in genetic research and breeding while considering the differences in race and virulence. It is also important to know the race or virulence level of strains in each production area to introduce the optimal resistant cultivars. To do this, the methods and materials used in this study can be utilized.

One of our most alarming results is that race 2 strains (IT21-1, IT21-2) that overcome *Ar-1*, which is a target gene in current watermelon breeding in Japan, are also present in Japan; these strains may cause severe damage to watermelon production. In addition, strains with high virulence to cucumbers, such as ST21-1, ST21-2 and MAFF 306737, which also cause severe damage even to C03 (showing the most resistance) and cultivars harboring *Cssgr*, are also present in Japan. Since these three strains also cause severe damage to the melon cultivars, they have the potential to be widespread and result in more destructive damage in open-field cultivation of a wide range of cucurbits in Japan. Therefore, it is important to identify resistance genes in the most resistant cultivar C03 and to accumulate resistance genes including *Cssgr* in future cucumber cultivars for stable resistance. Previous studies reported the existence of watermelon accessions with the *Ar-2* gene, which reduces damage by the race 2 strains (Boyhan *et al.* 1994, Guner and Wehner 2003). Therefore, it is necessary to introduce such genetic resources to Japan and to develop new cultivars harboring both *Ar-1* and *Ar-2*. Moreover, screening genetic resources for novel resistance genes using *C. orbiculare* strains with high virulence is of vital importance for watermelon, cucumber, and melon production.

## Author Contribution Statement

HM conducted experiments, analyzed data. YI and YK were supervised the experiment of single spore isolation. YY designed this study, supervised the experiments, and participated in data analysis. HM and YY wrote the manuscript with input from the other coauthors. All authors reviewed and approved this submission.

## Figures and Tables

**Table 1.** Reaction of watermelon hosts to *Colletotrichum orbiculare* strains distributed in Japan in a detached cotyledon assay

Strains	Designation	Source	Location	Co.*^a^*	AW*^b^*	AC*^c^*	Concentration (×10^6^ spores/mL)	Watermelon	
								W01 (‘Sugar Baby (Cocomero)’)	W02 (‘Charleston Gray’)
MAFF 306685	Y03010	watermelon	Yamagata	I	0	1	0.02	R	R
MAFF 306868	CL-3	cucumber	Saga	II	0	2	0.01	R	R
MAFF 240422	104-T	cucumber	Kyoto	II	0	2	4	R	R
MAFF 306518	MCK-1	melon	Miyagi	II	0	5	1.5	R	R
MAFF 237606	Kishi10(5)	cucumber	Tochigi	I	0	6	4	MR	MR
MAFF 243071	CoO-10	cucumber	Gunma	I	0	6	1.5	R	R
MAFF 306738	CcM-2	cucumber	Gunma	I	0	6	0.2	MR	R
MAFF 306795	S0520	cucumber	Aomori	II	0	6	1.5	MR	R
MAFF 306681	S0504	cucumber	Aomori	I	0	7	1.5	R	R
MAFF 306519	MCK-2	melon	Miyagi	II	1	4	0.2	S	MR
MAFF 242678	CO3	cucumber	Gunma	II	1	6	1.5	S	R
MAFF 306590	MCG-2	gerbera	Miyagi	II	1	6	1.5	S	R
MAFF 306870	CL-10	cucumber	Saga	II	1	6	1.5	S	R
A6	A6	watermelon	Nara	–	1	6	4	S	R
MAFF 726522	–	cucumber	Gunma	I	1	7	1.5	S	R
MAFF 306737	CcM-1	cucumber	Gunma	I	1	7	1.5	S	MR
ST21-2	ST21-2	cucumber	Saitama	II	1	7	1.5	S	MR
IT21-1	IT21-1	watermelon	Iwate	II	2	3	0.5	S	S
IT21-2	IT21-2	watermelon	Iwate	II	2	4	1.5	S	S
ST21-1	ST21-1	cucumber	Saitama	II	2	7	1.5	S	S

MR, moderately resistant; R, resistant; S, susceptible. *^a^* Co. is the phylogenetic group of *C. orbiculare* based on the ITS1-5.8S-ITS2 rDNA region. *^b^* AW is the group based on the reaction of watermelon hosts to *C. orbiculare*. *^c^* AC is the group based on the reaction of cucumber hosts to *C. orbiculare*.

**Table 2.** Reaction of cucumber hosts to *Colletotrichum orbiculare* strains distributed in Japan in a detached cotyledon assay

Strains	Co.*^a^*	AC*^b^*	AW*^c^*	Concentration (×10^6^ spores/mL)	Cucumber				
					C01 (PI 197087)	C02 (‘Poinsett’)	C03 (‘Ban Kyuri’)	C04 (‘B1’)	C05 (‘Tokiwa’)
MAFF 306685	I	1	0	0.02	MR	R	R	S	MR
MAFF 306868	II	2	0	0.01	S	MR	R	S	MR
MAFF 240422	II	2	0	4	S	MR	R	S	MR
IT21-1	II	3	2	0.5	MR	MR	R	MR	S
MAFF 306519	II	4	1	0.2	S	MR	MR	S	S
IT21-2	II	4	2	1.5	S	R	R	S	S
MAFF 306518	II	5	0	1.5	S	S	MR	S	MR
MAFF 237606	I	6	0	4	S	S	MR	S	S
MAFF 243071	I	6	0	1.5	S	S	R	S	S
MAFF 306738	I	6	0	0.2	S	S	R	S	S
MAFF 306795	II	6	0	1.5	S	S	MR	S	S
MAFF 242678	II	6	1	1.5	S	S	MR	S	S
MAFF 306590	II	6	1	1.5	S	S	MR	S	S
MAFF 306870	II	6	1	1.5	S	S	MR	S	S
A6	–	6	1	4	S	S	MR	S	S
MAFF 306681	I	7	0	1.5	S	S	S	S	S
MAFF 726522	I	7	1	1.5	S	S	S	S	S
MAFF 306737	I	7	1	1.5	S	S	S	S	S
ST21-2	II	7	1	1.5	S	S	S	S	S
ST21-1	II	7	2	1.5	S	S	S	S	S

MR, moderately resistant; R, resistant; S, susceptible. *^a^* Co. is the phylogenetic group of *C. orbiculare* based on the ITS1-5.8S-ITS2 rDNA region. *^b^* AW is the group based on the reaction of watermelon hosts to *C. orbiculare*. *^c^* AC is the group based on the reaction of cucumber hosts to *C. orbiculare*.

**Table 3.** Diversity of *Colletotrichum orbiculare* virulence to watermelon, melon, and squash in a seedling assay

Species	JP No.	Resistance gene	MAFF	240422	306518	243071	306681	242678	306590	306738	306795	237606	306519	306870	726522	306737	–	–	–	–
			Accession/ Strain	104-T	MCK-1	CoO-10	S0504	CO3	MCG-2	CcM-2	S0520	Kishi10 (5)	MCK-2	CL-10	–	CcM-1	ST21-2	ST21-1	IT21-1	IT21-2
			Co.*^a^*	II	II	I	I	II	II	I	II	I	II	II	I	I	II	II	II	II
			Race	0	0	0	0	0	0	1	1	1	1	1	1	1	1	1	2	2
			AW*^b^*	0	0	0	0	1	1	0	0	0	1	1	1	1	1	2	2	2
			AC*^c^*	2	5	6	7	6	6	6	6	6	4	6	7	7	7	7	3	4
Watermelon	32087	*ar-1/ar-1*	W01 (‘Sugar Baby (Cocomero)’)	2.0 bd	4.0 bf	2.6 bcd	2.3 cdef	4.3 ab	3.1 bcd	5.9 a	5.3 ac	4.6 bcde	5.8 ab	6.9 a	5.1 bce	6.7 a	7.0 a	7.0 a	7.0 a	7.0 a
Watermelon	32089	*Ar-1/Ar-1*	W02 (‘Charleston Gray’)	1.3 cd	0.6 gh	3.4 ab	1.4 eg	2.9 bc	1.6 ce	3.6 bc	3.3 ce	2.6 f	1.0 e	3.2 de	1.3 h	2.2 d	3.4 d	2.9 d	5.9 a	7.0 a
Watermelon	32091	*ar-1/ar-1*	W03 (‘Sugar Baby A’)	1.7 cd	2.8 def	2.2 bcd	1.6 dg	3.9 ac	3.6 ac	4.3 ac	4.1 ae	5.2 ac	6.4 a	6.1 ab	6.9 a	6.8 a	7.0 a	7.0 a	7.0 a	7.0 a
Watermelon		*ar-1/ar-1*	W04 (‘WF01’)	1.1 de	2.4 efg	2.9 bc	2.9 bcde	4.5 ab	1.6 ce	5.8 a	5.4 ac	6.4 a	5.7 ab	7.0 a	5.6 acd	7.0 a	7.0 ab	7.0 a	7.0 a	7.0 a
Watermelon		*Ar-1/ar-1*	W05 (‘WF02’)	1.1 de	0.4 gh	0.9 de	0.6 fg	1.9 cd	1.1 de	3.2 c	2.2 ef	2.8 f	1.0 de	3.0 e	1.6 gh	2.9 d	3.5 d	4.1 c	7.0 a	7.0 a
Melon		–	M01 (‘Earls Favorite Harukei 3’)	1.4 cd	4.1 be	2.9 bc	5.8 a	4.0 ac	4.5 ab	2.8 c	3.9 ae	4.6 bcde	7.0 a	7.0 a	6.5 ab	7.0 a	7.0 ab	7.0 a	6.2 a	6.6 a
Melon	91212	–	M02 (‘Edist 47’)	1.3 cd	6.6 a	3.4 bc	3.9 ac	3.8 ac	4.1 ab	5.7 a	3.2 de	4.6 bcde	6.0 ab	5.9 ab	6.6 ab	6.6 a	7.0 ab	7.0 a	5.4 ab	4.9 bc
Squash		–	S01	0.0 e	0.3 h	0.0 e	0.0 g	0.0 d	0.0 e	0.4 d	0.3 f	0.7 g	0.1 e	0.0 f	0.8 h	0.0 e	0.0 e	0.0 e	0.2 g	0.3 e

Within each line different letters indicate a significant difference in mean infection severity values (*P* < 0.05), based on Tukey HSD test. *^a^* Co. is the phylogenetic group of *C. orbiculare* based on the ITS1-5.8S-ITS2 rDNA region. *^b^* AW is the group based on the reaction of watermelon hosts to *C. orbiculare*. *^c^* AC is the group based on the reaction of cucumber hosts to *C. orbiculare*.

**Table 4.** Diversity of *Colletotrichum orbiculare* virulence to cucumber in seedling assay

JP No.	Resistance gene	MAFF	240422	–	243071	306681	306590	–	306518	306795	242678	237606	306738	306519	726522	306870	306737	–	–	Average IS score
		Accession/ Strain	104-T	IT21-1	CoO-10	S0504	MCG-2	IT21-2	MCK-1	S0520	CO3	Kishi10 (5)	CcM-2	MCK-2	–	CL-10	CcM-1	ST21-2	ST21-1	
		Co.*^a^*	II	II	I	I	II	II	II	II	II	I	I	II	I	II	I	II	II	
		Race	0	2	0	0	0	2	0	1	0	1	1	1	1	1	1	1	1	
		AC*^b^*	2	3	6	7	6	4	5	6	6	6	6	4	7	6	7	7	7	
		AW*^c^*	0	2	0	0	1	2	0	0	1	0	0	1	1	1	1	1	2	
138524	*Cssgr/Cssgr*	C01 (PI 197087)	1.6 cd	2.1 def	2.0 bcd	3.0 bcde	3.5 ac	3.7 c	4.5 bd	4.9 acd	4.2 ab	4.9 acd	5.2 ab	5.7 ab	6.6 ab	6.2 ab	7.0 a	7.0 a	7.0 a	4.7 ab
138528	*Cssgr/Cssgr*	C02 (‘Poinsett’)	1.6 cd	1.2 eg	1.9 bcd	2.3 cdef	3.2 acd	4.4 bc	3.5 bf	2.5 e	4.9 ab	3.9 cf	4.2 ac	4.5 bc	4.2 def	5.2 bc	5.9 ab	6.9 ab	6.8 a	3.9 ac
82352	*CsSGR/CsSGR^d^*	C03 (‘Ban Kyuri’)	1.4 cd	0.3 fg	1.5 ce	1.8 cdg	2.6 bcd	1.6 de	2.1 fh	3.1 de	2.9 bc	3.0 ef	3.0 c	3.0 cd	3.1 fg	3.6 ce	4.4 c	4.8 c	5.0 b	2.8 c
	*CsSGR/CsSGR*	C04 (‘B1’)	4.0 a	3.9 bc	4.9 a	4.9 ab	5.1 a	4.7 bc	5.2 abc	6.0 a	5.9 a	5.7 ab	6.0 a	5.7 ab	6.0 ac	6.0 ab	6.0 ab	6.4 ab	7.0 a	5.5 a
83555	*CsSGR/CsSGR*	C05 (‘Tokiwa’)	1.0 de	2.7 ce	1.8 bcd	3.0 bcde	2.7 bcd	3.3 cd	3.9 bf	2.6 e	3.2 bc	3.4 df	3.3 bc	6.0 ab	4.7 cf	5.4 ac	6.4 a	7.0 a	7.0 a	4.0 ac
32344	*Cssgr/Cssgr*	C06 (‘Marketer’)	1.3 cd	1.3 eg	1.8 be	1.6 cdg	2.9 bcd	1.2 e	3.4 cdef	3.7 bce	4.3 ab	3.3 df	4.8 ac	3.6 c	3.6 ef	4.9 bcd	4.9 bc	5.9 b	6.5 a	3.5 bc
216082	*CsSGR/CsSGR*	C07 (‘Model’)	3.0 ab	4.1 bc	2.9 bc	3.8 ad	4.3 ab	5.6 ab	5.4 ab	5.5 ab	4.9 ab	5.5 ac	5.8 a	6.1 ab	6.9 a	7.0 a	6.7 a	7.0 ab	7.0 a	5.4 a
216085	*CsSGR/CsSGR*	C08 (‘SMR58’)	2.4 bc	3.4 cd	2.9 bc	3.1 bcde	3.6 ac	4.4 bc	4.3 be	4.0 ae	4.3 ab	4.9 acd	4.5 ac	4.4 bc	5.8 ac	5.7 ab	6.7 a	7.0 ab	7.0 a	4.6 ab

Within each line different letters indicate a significant difference in mean infection severity values (*P* < 0.05), based on Tukey HSD test. *^a^* Co. is the phylogenetic group of *C. orbiculare* based on the ITS1-5.8S-ITS2 rDNA region. *^b^* AC is the group based on the reaction of cucumber hosts to *C. orbiculare*. *^c^* AW is the group based on the reaction of watermelon hosts to *C. orbiculare*. *^d^* Different resistance gene from *Cssgr*.
